# Drug Development for the Irritable Bowel Syndrome: Current Challenges and Future Perspectives

**DOI:** 10.3389/fphar.2013.00007

**Published:** 2013-02-01

**Authors:** Fabrizio De Ponti

**Affiliations:** ^1^Department of Medical and Surgical Sciences, University of BolognaBologna, Italy

**Keywords:** drug targets, translational medical research, brain-gut interactions, drug selectivity, biomarkers, 5-hydroxytryptamine, transient receptor potential channels, neuroimmune intestinal interactions

## Abstract

Medications are frequently used for the treatment of patients with the irritable bowel syndrome (IBS), although their actual benefit is often debated. In fact, the recent progress in our understanding of the pathophysiology of IBS, accompanied by a large number of preclinical and clinical studies of new drugs, has not been matched by a significant improvement of the armamentarium of medications available to treat IBS. The aim of this review is to outline the current challenges in drug development for IBS, taking advantage of what we have learnt through the Rome process (Rome I, Rome II, and Rome III). The key questions that will be addressed are: (a) do we still believe in the “magic bullet,” i.e., a very selective drug displaying a single receptor mechanism capable of controlling IBS symptoms? (b) IBS is a “functional disorder” where complex neuroimmune and brain-gut interactions occur and minimal inflammation is often documented: do we need to target gut motility, visceral sensitivity, or minimal inflammation? (c) are there validated biomarkers (accepted by regulatory agencies) for studies of sensation and motility with experimental medications in humans? (d) do animal models have predictive and translational value? (e) in the era of personalized medicine, does pharmacogenomics applied to these medications already play a role? Finally, this review will briefly outline medications currently used or in development for IBS. It is anticipated that a more focused interaction between basic science investigators, pharmacologists, and clinicians will lead to better treatment of IBS.

## Introduction

Irritable bowel syndrome (IBS) is a common functional gastrointestinal disorder, characterized by recurrent abdominal pain or discomfort in combination with disturbed bowel habits in the absence of identifiable organic cause. Many medications are used for the treatment of patients with IBS, although their actual benefit is often a matter of debate. In particular, only a few are specifically labeled for IBS. In fact, notwithstanding great progress in our understanding of the pathophysiology of IBS thanks to a large number of preclinical and clinical studies of new drugs, the specific armamentarium of medications available is scant. The aim of this review is to outline the current challenges in drug development for IBS, taking advantage of what we have learned through the Rome process (from Rome I in the 1980s to Rome III published in 2006; Drossman, 2006).

The key questions that will be addressed are: (a) do we still believe in the “magic bullet,” i.e., a very selective drug displaying a single receptor mechanism capable of controlling IBS symptoms? (b) IBS is a “functional disorder” where complex neuroimmune and brain-gut interactions occur and minimal inflammation is often documented: do we need to target gut motility, visceral sensitivity, or minimal inflammation? (c) are there validated biomarkers (accepted by regulatory agencies) for studies of sensation and motility with experimental medications in humans? (d) do animal models have predictive and translational value? (e) in the era of personalized medicine, does pharmacogenomics applied to these medications already play a role? Finally, this review will briefly outline medications currently used or in development for IBS. It is anticipated that a more focused interaction between basic science investigators, pharmacologists, and clinicians will lead to better treatment of IBS.

## The “Magic Bullet”: A Concept That Needs Rethinking

The key pharmacodynamics, pharmacokinetic, and safety features for drugs to be used in the treatment of IBS are outlined in Table 1.

**Table 1:** **Key features for drugs to be used in the treatment of IBS**.

	Key features
Pharmacodynamics	The drug should target a whole pathophysiological mechanism rather than a single receptor
	*Possible targets: motility, secretion, visceral sensitivity, neuroimmune interactions/minimal inflammation, brain-gut axis*
	The effect should be maintained over time during treatment
Pharmacokinetics	Good oral bioavailability (unless local action in the gut is specifically wanted)
	Half-life allows once daily dosing
	No metabolites with different or unwanted pharmacological actions
	Avoid CYP substrates with high likelihood of drug interactions
	Consider interactions with food or herbal products
Safety	Specificity cannot always avoid *off-target* effects because the same receptor/system also mediates other effects
	A drug can also hit *antitargets* (i.e., unwanted targets), another source of side-effects

A selective drug is defined as a compound interacting only with one receptor subtype and leaving other receptors unaffected at concentrations achieved at therapeutic doses. The literature on the treatment of IBS has often resorted to the concept of the “magic bullet,” i.e., a very selective drug displaying a single receptor mechanism capable of controlling IBS symptoms (Camilleri et al., 2006a). This was often considered the key to efficacy avoiding side-effects. This approach is no longer ideal because of several important pitfalls.

First, drug selectivity is always a relative concept, which ignores the basic fact that most molecules, even at therapeutic doses, may have several, sometimes disparate biological effects (i.e., hit a large number of targets in the pharmacological space; Garcia-Serna et al., 2010; Kawasaki and Freire, 2011). These effects may depend on the fact that a single receptor/effector pathway plays a role in different systems, so that even selective compounds have *off-target* effects (Table 1). In addition, there are many instances when the compound is endowed with additional pharmacological properties that hit the so-called *antitargets* (i.e., unwanted targets), responsible for side-effects, which are clarified only after the compound has undergone clinical trials. The classical example is provided by the cardiac side-effects due to hERG K^+^ channel blockade by the early 5-HT_4_ receptor agonists (Tonini et al., 1999).

The second issue is that the multifactorial pathophysiology of IBS (with multiple brain-gut and neuroimmune interactions) makes it unrealistic to expect that drugs acting on a single receptor may achieve substantial therapeutic gain over placebo in an area where the placebo response rate is substantial (approaching 40% across all randomized controlled trials; Ford and Moayyedi, 2010). As in other fields (Morphy et al., 2004), evidence suggests that a balanced modulation of multiple targets can provide a superior therapeutic effect and side effect profile compared to the action of a selective ligand. *Designed* multiple ligands that hit a large variety of targets have been produced through rational approaches in which structural features from selective ligands are combined (Morphy et al., 2004). A key challenge in the design of multiple ligands is attaining a balanced activity at each target of interest with a suitable pharmacokinetic profile.

The third issue is that mechanisms underlying symptoms in IBS may differ among patients, hence the need to consider using multiple therapies. With selective drugs, primary clinical endpoints were achieved in less than 70% of patients with the approved agents such as tegaserod or alosetron (Camilleri et al., 2000; Muller-Lissner et al., 2001; Cremonini et al., 2003). On the other hand, it seems reasonable to propose treatment with combination therapy, which is the rule when treating medical conditions such as hypertension or asthma, when monotherapy is no longer adequate. Because of the redundancy of mechanisms controlling neurosensory, neuromuscular, and neuroimmune functions in the gut, it is conceivable that effective treatment of functional gut disorders may require combination therapy.

One example is provided by tachykinin receptor antagonists, which have so far given disappointing results because of inherent differences among animal models and humans: it has been suggested that the analgesic efficacy of multi- or pan-tachykinin receptor antagonists is superior to that of mono-receptor antagonists (Holzer, 2004a).

When drugs address a specific target (e.g., a symptom such as visceral hypersensitivity or motility), heterogeneity in the pathophysiology impacts negatively on the therapeutic gain, if patients are not carefully selected in a clinical trial. Indeed, some of the disappointing results of the past can be ascribed to the lack of understanding of pathophysiology: the same symptom (e.g., diarrhea) does not necessarily depend on the same pathways in all patients.

Thus, new drugs should target a pathophysiological mechanism (provided that it is known!), rather than a specific receptor; on the other hand, recruiting carefully selected patient subgroups may significantly reduce the generalizability of the results of the trial.

Pharmacokinetics may help to achieve gut selectivity and reduce side-effects. This approach is particularly relevant when there are potential actions outside the gut, as it is indeed the case with peripherally restricted opioid receptor antagonists (such as methylnaltrexone and alvimopan), which do not cross the blood brain barrier and, in addition, have very low oral bioavailability (De Ponti, 2002; Holzer, 2004b). An example of minimally absorbed compound is also the guanylate cyclase-C agonist linaclotide (Wensel and Luthin, 2011; Busby et al., 2013), which is now FDA- and European Medicines Agency (EMA)-approved for IBS with constipation.

Another important pharmacokinetic property is the lack of interactions with food or other drugs. Significant interactions with CYP2D6 and CYP3A4 should be predicted in early drug discovery because of their involvement in drug metabolism with important pharmacogenetic aspects.

Finally, as regards safety aspects, apart from the standard safety evaluations, two issues deserve special attention following the experience with cisapride (torsades de pointes associated with QT prolongation; De Ponti et al., 2001) and alosetron (ischemic colitis; Moynihan, 2002). It is clear that even very rare events may negatively impact the risk/benefit balance of drugs that are used to provide symptom improvement of non-serious (though troublesome) diseases such as IBS (De Ponti et al., 2002; Tack et al., 2012). It is remarkable that in IBS with diarrhea, Shah et al. (2012) found that one adverse event resulting in study discontinuation occurred for every 2.3 and 2.6 patients who benefited, respectively, from tricyclic antidepressants and alosetron, i.e., the number needed to harm was approximately 3. This is quite low, considering the numbers needed to treat reported in the literature for drugs in IBS (Brandt et al., 2009; Camilleri and Mayer, 2009; Menees et al., 2012). Shah et al. (2012) conclude that, rather than simply focusing on the number needed to treat, clinicians should be aware of harm when using pharmacotherapy for IBS.

## IBS as a “Functional Disorder”: New Perspectives and Global Regulatory Framework

The classical concept of IBS as a functional disorder derives from the fact that no organic cause can be identified and the diagnosis of IBS is one of exclusion after other disorders have been ruled out. In addition, the precise mechanisms underlying symptom generation are unknown.

However, research of the past 20 years has provided significant advances in the understanding of the pathophysiology of IBS, with an emerging consensus that the various clinical manifestations (including non-gastrointestinal comorbid symptoms) of chronic abdominal pain can best be viewed as a dysregulation in the complex interplay between events occurring in the gut lumen (including microbiota), the mucosa, the enteric nervous system (ENS), and the central nervous system (Mayer and Tillisch, 2011; Matricon et al., 2012). This dysregulaton leads to alterations in sensation, motility, brain-gut interactions, and neuroimmune interactions. Considerable evidence documents that sensitizing proinflammatory mediators, mast cells and their products, tryptases, are increased in tissues of patients with colorectal hypersensitivity (Cenac et al., 2007; Balestra et al., 2012; Buhner et al., 2012).

It has been shown that colonic mast cell infiltration and mediator release in proximity to mucosal innervation may contribute to abdominal pain perception in IBS patients (Barbara et al., 2004). Indeed, mucosal mast cell mediators from IBS patients excite rat nociceptive visceral sensory nerves (Barbara et al., 2007). In a recent study (Balestra et al., 2012), mucosal biopsies were obtained from the descending colon of patients with IBS and controls. Mucosal mast cells were identified immunohistochemically. The impact of spontaneously released mucosal mediators on guinea pig electrically stimulated longitudinal muscle myenteric plexus (LMMP) preparations was assessed *in vitro* by means of selective receptor antagonists and inhibitors. Patients with IBS showed an increased mast cell count compared with controls. Application of mucosal mediators of IBS to LMMPs potentiated cholinergic twitch contractions, an effect directly correlated with mast cell counts and mediated by activation of prostanoid receptors, TRPV1, and P2X receptors. These results support the role of mucosal inflammatory mediators and mast cell activation in altered motor function of IBS.

It is also intriguing that, in patients with IBS, 5-HT spontaneous release was significantly increased irrespective of bowel habit and correlated with mast cell counts and the severity of abdominal pain. This suggests that increased 5-HT release contributes to development of abdominal pain in IBS, probably through mucosal immune activation (Cremon et al., 2011).

Several studies have reported the onset of IBS-like symptoms following established bacterial or viral infections of the GI tract (Barbara et al., 2009). This so-called “postinfectious” IBS occurs in approximately 10% of patients undergoing a documented infectious gastroenteritis, and risk factors to develop symptom persistence are longer duration of the gastroenteritis, female sex, psychosocial stressors at the time of the infection, and psychological factors such as anxiety or depression. Although a causal relationship between abdominal pain and acute or chronic infections cannot be established most of the times, it is tempting to speculate that host-microbial interactions in vulnerable individuals during the early phase of the disorder may lead to permanently altered immune response, which then continues to play a role when symptoms persist in the absence of the infectious organism.

The participation of the gut microenvironment in the pathophysiology of IBS is suggested by studies indicating an interplay between luminal factors including the microbiome, the epithelial barrier, and the mucosal immune system (Stanghellini et al., 2010; Camilleri et al., 2012). In an animal model (McVey Neufeld et al., 2013), microbiota were shown to be necessary for normal excitability of gut sensory neurons and this provides a potential mechanism for the transfer of information between the microbiota and the nervous system.

In postinfectious IBS and in IBS with diarrhea, decreased expression and structural rearrangement of tight junction proteins in the small bowel and colon may lead to increased intestinal permeability. These abnormalities might contribute to the outflow of antigens through the epithelium, causing overstimulation of the mucosal immune system. Accordingly, subgroups of patients with IBS show higher numbers and activation of mucosal immunocytes, especially mast cells. Immune factors, released by these cells, including proteases, histamine, and prostanoids, might also participate in maintaining the permeability dysfunction and contribute to the activation of abnormal neural responses, which, in turn, are involved in abdominal pain perception and changes in bowel habits.

All these mechanisms represent new therapeutic targets in IBS. Here, it is important to remember that probiotics are also currently viewed as an attractive therapeutic option in IBS because of their recognized safety and of their documented biological effects on the host. Preclinical studies have shown that some probiotic strains exhibit potentially useful properties including anti-inflammatory effects, improvement of mucosal barrier homeostasis, beneficial effects on intestinal microbiota, and a reduction of visceral hypersensitivity. However, it remains to be determined to what extent a beneficial effect on these parameter translates to a significant effect on clinical outcomes: although the effect of probiotics on IBS is positive in some randomized, controlled studies, the gain over placebo is small and identification of a tailored probiotic approach for subgroups of patients represents a future challenge.

The complex neuroimmune and brain-gut interactions sometimes associated with minimal mucosal inflammation (Ford and Talley, 2011) and neuroplastic changes in the ENS (Giaroni et al., 1999) pose several questions as regards potential targets for pharmacological intervention: should the therapeutic focus be primarily gut motility, visceral sensitivity, or minimal inflammation? Assessment of these parameters in humans can be undertaken by using a variety of invasive and non-invasive techniques, some well established and others requiring further validation. By using these techniques, alterations in both sensory and motor function have been reported in IBS and our understanding of sensorimotor dysfunction has indeed increased. Thus, inflammatory, immunologic, and other processes, as well as psychosocial factors such as stress, can alter the normal patterns of sensitivity and motility through alterations in local reflex activity or via altered neural processing along the brain-gut axis. A firm relationship between sensorimotor dysfunction and the production of symptoms, however, has been difficult to show. Thus, the clinical relevance of the former requires further research.

In this context, it is important to remember that in 2003 the EMA adopted a document produced by the Efficacy Working Party on the “*Points to consider on the evaluation of medicinal products for the treatment of the irritable bowel syndrome*” (EMA, 2003). Although the document now needs to be updated (it still refers to Rome II criteria), a key statement is that “*The patient’s global assessment of symptoms and abdominal discomfort/pain should be used as the two primary endpoints. Statistically significant changes must be found in both parameters*.” Thus, clinical efficacy must rely on clinical endpoints in the patient’s perspective, for instance through the global assessment of multiple symptoms. Mechanistic (pathophysiological) studies provide a rationale for drug development, but do not generally predict symptomatic success and do not necessarily identify the most appropriate dose for clinical trials. An important goal is to develop non-invasive tests that identify important pathophysiological mechanisms and assess symptom pattern in short-term (4 weeks) therapeutic trials that pave the way to longer trials. Notably, the EMA document carefully considers the duration of efficacy trials, stating that they must be long enough to determine whether the response is sustained and to determine the effects of treatment withdrawal. A duration of 6 months of active treatment is considered necessary considering the cyclic and non-life threatening nature of the disease.

For inclusion and exclusion criteria for IBS, the current EMA “*Points to consider*” document refers to Rome II criteria (current Rome III criteria differ from Rome II and, notably, the Rome IV process is expected to start in 2013). A revised EMA guideline is awaited soon and should come into force by the end of 2013. A key issue that needs to be addressed, apart from the update to Rome III, is the discrepancy with the FDA guidance issued in 2012 (FDA, 2012). Indeed, the EMA document recommends the two co-primary endpoints indicated above, whereas the FDA guidance recommends a primary endpoint that measures the effect of treatment on two major IBS signs and symptoms: abdominal pain and abnormal defecation (stool frequency or stool consistency, depending on subtype of IBS). The FDA guidance also acknowledges that patient-reported outcome (PRO) measures of the signs and symptoms of the condition are the only currently available measures that can adequately define a treatment effect in a clinical trial. In addition, because of the limitations of using a single-item patient-reported rating of overall change as a primary endpoint, the FDA document recommends the development of a multi-item PRO instrument. The PRO measure(s) should capture all the clinically important signs and symptoms of the target population. The ongoing regulatory discussion will certainly help all those involved in clinical trials to plan future research.

Linaclotide may serve as an example of the current regulatory situation, with differences in endpoints recommended by the FDA and the EMA: the efficacy and safety of this agent in patients with IBS-C was evaluated in two randomized, placebo-controlled Phase 3 trials. These trials were designed according to both FDA and EMA guidelines and findings based on FDA-recommended endpoints were reported in two recent studies (Chey et al., 2012; Rao et al., 2012), whereas the findings of a planned, separate analysis of both trials based on the distinct efficacy parameters prespecified for EMA submission were published separately (Quigley et al., 2013).

In closing this section, it should be remembered that, over the past 25 years, the Rome process has insisted on clinical features to diagnose IBS on the assumption that grouping of patients with similar features facilitates identification of patients most likely to respond to a given pharmacological agent, but this is not necessarily so because the same symptom (pain) may have several underlying pathophysiological mechanisms. This explains the criticism raised by some investigators against the Rome criteria (Dang et al., 2012). On the other hand, it must be acknowledged that there are several reasons to establish an accurate diagnosis of IBS: to relieve patient uncertainty and initiate the most appropriate treatment, avoiding the burden of unnecessary medications or diagnostic procedures and surgeries (Mearin and Lacy, 2012). In other words, the Rome criteria try to transform the diagnosis of IBS from one of exclusion into a positive diagnosis based on history, physical examination, use of precise diagnostic criteria in the absence of specific alarm features.

## Biomarkers for IBS

Biomarkers are objectively measurable indicators of normal or pathological processes or pharmacological responses to a therapeutic intervention (Anonymous, 2001). In order to improve development and usage of biomarkers, a score system of different types of biomarkers has been proposed, depending on their impact on drug development (Wehling, 2009).

Positive modifications of biomarkers which imply improvement of the disease can be taken as endpoints during drug development. To provide a more complete picture, a therapeutic target can coincide with the biomarker (e.g., TNFα) or, as a component of the disease mechanism, modulate it (e.g., NF-κB).

Unfortunately, as stated in the 2012 FDA document, no validated and accepted biomarker exists in IBS. In addition, the limited repertoire of clinical manifestations of sensorimotor disorders of the gut such as IBS can actually derive from multiple mechanisms, leading in turn to similar symptoms. In many clinical programs of new drugs for IBS, the emphasis was primarily on symptom assessment of broad groups of patients identified by the Rome criteria. As already discussed above, this approach was not ideal and it not surprising that drugs of potential value have been abandoned.

Certain biomarkers can, in a limited fashion, be used to predict the success of a drug in IBS or to understand its mode of action. These studies may be incorporated in the recommended steps for drug development, but should be viewed only as preliminary/complementary steps of the development program, which must comply with the regulatory guidance quoted in the previous section.

Currently established tests that can be used as potential biomarkers for clinically relevant endpoints in IBS include the following.

### Intraluminal measurements of colonic or rectal motility and sensation

Intraluminal measurements may serve as biomarkers for motor or sensory modulation in IBS. Manometry has long been used for pathophysiological investigations (Camilleri et al., 2008) and can be used as a useful technique to study the effects of drugs on colonic motility (De Schryver et al., 2002; Dinning and Scott, 2011). Another possible test is intracolonic measurement of postprandial tone using a barostat, which, in healthy subjects (von der Ohe et al., 1994), showed the potential of 5-HT_3_ receptor antagonists to prevent diarrhea and other postprandial symptoms in diseases including IBS and carcinoid diarrhea (von der Ohe et al., 1993). This indicates that measuring tone intraluminally may be a useful biomarker for preliminary tests before subsequent trials for efficacy. A recent study in healthy subjects (Sweetser et al., 2009) investigated the effects of lubiprostone on colonic sensation and motility with the following endpoints: colonic compliance, fasting and postprandial tone and motility indexes, pain thresholds, and sensory ratings to distensions. This investigation well exemplifies the potential of pharmacodynamic studies in drug development.

Although testing visceral sensitivity may provide useful mechanistic insights when developing new medications, results always require careful interpretation and are sometimes disappointing. For instance, alosetron was shown to alter colonic compliance, but not colonic sensitivity to isobaric distension (Delvaux et al., 1998). Previously, the κ opioid receptor agonist fedotozine was shown to decrease sensitivity to colonic distension, but the therapeutic gain in placebo-controlled studies in IBS was found to be of insufficient magnitude for further development (Dapoigny et al., 1995; Delvaux et al., 1999; Ness, 1999). Asimadoline is another example of drug tested for its effect on visceral sensitivity in humans (Delgado-Aros et al., 2003).

One disadvantage of sensation biomarkers is that the sample size required to avoid a type 2 error while assessing clinically meaningful effect sizes is higher than with transit endpoints in healthy volunteers and probably even higher in patients. Nevertheless, these sample sizes (12–20 per treatment arm) are still more practical than testing symptom endpoints, which require much larger samples. Thus, a 25–30% effect size can be demonstrated with ∼20 subjects per treatment arm in sensation-based studies and ∼12 per treatment arm in studies of transit, on the basis of the variability reported in published studies (Camilleri et al., 2006b).

### Radiopaque markers for colonic transit

The radiopaque marker test for colonic transit is a widely available test, as shown by early studies with loperamide for diarrhea and fiber for constipation, where radiopaque markers were used to assess whole gut transit time (Cann et al., 1984a,b). The overall effects of drugs for IBS can be predicted by the marker transit test, although transit times are not characteristic of IBS (Horikawa et al., 1999) and other studies addressing more specific endpoints suggest that the colonic marker transit time (<15 or >60 h) accurately predicts the extremes of stool consistency, with significant overlap for transit times between those extremes (Degen et al., 2001).

### Radioscintigraphic markers for colonic transit

Scintigraphic transit measurements are sufficiently well characterized to allow meaningful pharmacodynamic conclusions on the effect of therapeutic agents (Cremonini et al., 2002; Camilleri, 2010a; Vazquez-Roque et al., 2012). Namely, several examples support the use of detailed colonic transit measurement in the development of medications for IBS-associated changes in bowel function. First, alosetron (a 5-HT_3_ receptor antagonist) slows overall colonic transit and, on average, increases the time for emptying the ascending colon by 50% (Viramontes et al., 2001a). Second, tegaserod (a 5-HT_4_ receptor agonist) accelerates overall colonic transit and, on average, halves the time for emptying the ascending colon (Prather et al., 2000), and several studies showed that this medication was effective in the treatment of IBS with constipation (Muller-Lissner et al., 2001; Novick et al., 2002; Kellow et al., 2003). However, tegaserod was withdrawn by the manufacturer for safety issues in 2007 and was made available only under a restricted access program (Al-Judaibi et al., 2010). Finally, a more recent example of use of scintigraphy to assess transit includes linaclotide (Andresen et al., 2007).

### Urine sugars for *in vivo* gut permeability

Because of the possible role of disruption of intestinal mucosal barrier function in the pathophysiology of IBS, recently a urine sugar (lactulose and mannitol) excretion test was validated in patients with IBS and diarrhea (Rao et al., 2011). Urine sugars at 0–2 and 8–24 h reflect small bowel and colonic permeability, respectively and are increased in patients with IBS and diarrhea vs. controls. This method can be applied to study the effects of agents directed at mucosal pathophysiology, such as mast cell stabilizers or modulators of microbial flora.

## Animal Models: Predictivity Issues and Current Role

Translational medicine defines conditions and prerequisites to transfer more reliable results obtained from preclinical biomedical research to clinical applications, thus improving patient care through timely and efficient promotion of clinical innovation.

A disease model should mimic the clinical disease condition as much as possible and allow the investigation of unclear pathophysiological mechanisms toward the development of new potential therapeutic options. In spite of undeniable differences among species, animal models still represent the major source of information about biological system and provide an invaluable means to study complex physiological and biochemical interactions.

The predictivity of the disease model itself will substantially depend on the efforts toward optimization and scientific validation of the model, both in terms of resemblance and transferability to humans. Pharmacological targets and biomarkers must be reproduced and be susceptible to pharmacological modulation in the animal model, to ensure that the model is predictive of a therapeutic effect in humans. The real translational value of a biomarker is represented by its capacity of being thoroughly informative after its validation in clinical studies which provide the bedside to bench approach.

An animal model used as a screening tool in drug development should ideally reproduce different biomarkers providing multiple endpoints to fully assess the spectrum of activity of a test compound. Accurate selection of endpoints plays a pivotal role to assure the translational value of disease models and should take into account the sensitivity and specificity of each endpoint toward prediction of activity.

Although development of new drugs for treatment of IBS can be facilitated by preclinical animal models (Bulmer and Grundy, 2011; Holschneider et al., 2011), it must be acknowledged that a single sufficiently predictive model of efficacy still does not exist and investigators have the option to use multiple models to assess different aspects of the pathophysiology of IBS. This section is only intended as a brief outline of the most commonly used animal models of visceral pain and disturbed gastrointestinal motility, which are reviewed elsewhere (Camilleri et al., 2006a).

### Motility

The techniques used to record motility or measure transit in animals may differ from techniques used in humans but the endpoints are identical to those mentioned in the previous section.

### Visceral pain

There are several forms of stimulation and endpoints to measure visceral pain: (a) *mechanical stimuli*: experiments can be performed in awake or anesthetized rats, and the most frequently used stimulus of pain in animals is distension of a gut segment with a balloon connected to a barostat to measure simultaneously compliance and the response to the painful stimulus (Rouzade et al., 1998). Poor standardization of methods across laboratories is a drawback of these investigations. (b) *Chemical stimuli:* infusing glycerol into the rat colon through a chronically implanted catheter induces abdominal cramps. This model is considered relevant because intracolonic glycerol induces abdominal pain in humans and mimics pain reported by patients with IBS (Louvel et al., 1996). (c) *Other stimuli:* the significance of other models of visceral pain, such as the “writhing test” (consisting of an intraperitoneal injection of an irritant compound such as acetic acid), is questionable.

In the aforementioned models, it is important to distinguish between evaluation of *allodynia* (decrease in the threshold of sensitivity to distension) and *hyperalgesia* (enhanced response to painful stimulus). A commonly used endpoint is the contraction of abdominal muscles induced by rectal or colorectal distension in the rat; the contractions are recorded by electromyography, where the number of spike bursts correlates with the intensity of the stimulus applied (Morteau et al., 1994).

Visceral distension also induces *viscero-visceral reflexes*, such as relaxation of anal sphincters during rectal distension or change in *blood pressure*, which is a pseudoaffective response used to assess visceral pain.

In summary, selection of one or more definitive animal models of visceral hyperalgesia is not possible and using results from more than one animal model may enhance the probability of selecting effective drugs for further development. Since pain, though a major problem, is not the only symptom affecting quality of life, animal models detailing the effects of drugs on motility and visceral sensitivity may add a further dimension to the assessment of new compounds.

## The Role of Pharmacogenomics

Pharmacogenomics refers to the variability of the expression of individual genes relevant to disease susceptibility as well as drug response at cellular, tissue, individual, or population level. Pharmacogenetics refers more specifically to the study of individual variations in DNA sequence related to drug response. The growing interest in this field is due to the fact that risk/benefit evaluations of drugs are not fully appreciated if one does not fully consider individual variations that may significantly affect pharmacokinetics and pharmacodynamics.

### Polymorphisms as markers of disease

As an example, patients with IBS had significantly reduced frequencies of the high producer genotype for interleukin 10 than controls (21 vs. 32%; *p* = 0.003): this suggests a genetic predisposition in at least some patients with IBS to produce lower amounts of the anti-inflammatory cytokine interleukin 10 (Gonsalkorale et al., 2003) and lends support to the hypothesis that there may be an inflammatory or genetic component in some cases of IBS (Bashashati et al., 2012).

### Polymorphisms affecting drug response

Genetic variations can affect drug response in at least three different ways (Camilleri and Katzka, 2012): (a) changes in drug metabolism, e.g., through functional CYP450 2D6 genes, which determine the pharmacokinetics and plasma levels of many commonly used agents such as antidepressants; (b) changes in drug transporters, which may affect the response to medications: for instance, polymorphisms in the promoter for synthesis of serotonin transporter (SERT-P) influence response to serotonergic medications in depressed individuals. SERT polymorphisms were associated with a greater colonic transit response in those with long homozygous than those with heterozygous polymorphisms in D-IBS (Camilleri et al., 2002); (c) genetic polymorphism in drug targets. Several examples are provided by recent studies (Camilleri and Katzka, 2012; Vazquez-Roque et al., 2012).

In summary, pharmacogenetics is a rapidly growing field which may provide important pieces of information to fully understand the variability of drug action in patients and cannot be ignored in drug development programs, although its exploitation probably still needs some time.

## Classes of Drugs Used or Under Development in IBS

A detailed discussion of all the classes of drugs proposed as potential therapeutic agents in IBS is beyond the scope of this review. The main pharmacological approaches to IBS are summarized in Table 2, which does not include agents traditionally used in IBS, such as laxatives and antidiarrheal agents, respectively for constipation and diarrhea, and probiotics. The gut microbiome as a therapeutic target is covered elsewhere (Floch et al., 2011; Simren et al., 2013). The reader is also referred to a recent detailed review of current and potential pharmacological approaches in IBS (Camilleri, 2012). Herbal preparations used in IBS are covered by another review (Rahimi and Abdollahi, 2012). Brain-gut interactions and possible sites/mechanisms of pharmacological intervention along the brain-gut axis are discussed in a recent review in this Journal (Fichna and Storr, 2012).

**Table 2:** **Main pharmacological approaches in IBS**.

Drug class	Examples	Pharmacodynamics
**5-HT_4_ receptor agonists**	Prucalopride	Enteric neurons, smooth muscle cells
	Naronapride	Increased motility/bowel frequency
	Velusetrag	
	TD-8954	
**5-HT_3_-receptor antagonists**	Ramosetron	Inhibition of secretion, motility, nociception
**TPH_1_ blocker**	LX-1031	Inhibits 5-HT synthesis by blocking tryptophane hydroxylase 1
**Cl-C2 channel activator**	Lubiprostone	Increased intestinal water and electrolyte secretion
		Accelerates transit
**Guanylate cyclase-C agonist**	Linaclotide	Increased intestinal water and electrolyte secretion
		Accelerates transit
**PAR2 blockers**	GB88	Inflammation
**TRPV1 blockers**	Capsazepine	Inflammation
**TRPV4 blockers**	RN1734	
**Mast cell stabilizers**	Ketotifen	Inflammation
**μ-Opioid receptor agonists**	Loperamide	Enterocyte, enteric neurons, afferent neurons, and inflammation
**μ-Opioid receptor antagonists**	Naloxone	Enteric neurons, afferent neurons, and inflammation
	Methylnaltrexone alvimopan	
**κ-Opioid receptor agonists**	Asimadoline	Enteric neurons and afferent neurons
		Increase in sensory threshold
**β_3_-Adrenoceptor agonists**	Solabegron	Smooth muscle
**α_2_-Adrenoceptor agonists**	Clonidine	Enteric neurons and enterocytes
**NK_1_ receptor antagonists**	Ezlopitant	Enteric neurons, ICC, smooth muscle, immune cells
**NK_2_ receptor antagonists**	Nepadutant	Reduced smooth muscle contractility
**NK_3_ receptor antagonists**	Talnetant	Role in nociception not confirmed in clinical trials in patients with IBS
**CCK_1_ receptor antagonists**	Loxiglumide	Afferent vagal nerves and enteric neurons
**Antibiotics**	Rifaximin	Poorly absorbed with virtually no systemic effects

Several meta-analyzes of pharmacological treatments for IBS have been published in recent years and there is ongoing debate on their interpretation (Lesbros-Pantoflickova et al., 2004; Brandt et al., 2009; Camilleri and Mayer, 2009).

### Serotonergic agents

5-Hydroxytryptamine (5-HT) plays a key role in the control of gastrointestinal motility, sensitivity, and secretion (De Ponti, 2004). Several 5-HT receptor types are present on both nerves and smooth muscle and mediate a number of different actions (De Ponti, 2004). Actions of 5-HT are terminated by a reuptake system, which is inhibited by antidepressants (Gershon and Jonakait, 1979). *Selective serotonin reuptake inhibitors* (SSRIs) alter motility in the stomach, small bowel, and colon (Gorard et al., 1994), but no convincing beneficial therapeutic effects have been reported in IBS. Interestingly, the tryptophane hydroxylase inhibitor LX-1031 was recently reported to have therapeutic potential in IBS (Tack et al., 2011).

*5-HT_3_ receptor antagonists* include alosetron and ramosetron (Camilleri et al., 2000; Hirata et al., 2007; Lee et al., 2011); alosetron delays orocecal and colonic transit times, and reduce colonic compliance but not sensitivity to isobaric distension (Gore et al., 1990; Talley et al., 1990; Scolapio et al., 1995). Shortly after its introduction, alosetron was withdrawn due to suspected side-effects of colonic ischemia (Moynihan, 2002).

*5-HT_4_ receptor agonists*, after withdrawal of tegaserod, now include prucalopride, naronapride, velusetrag (Camilleri, 2010b), and TD-8954 (Beattie et al., 2011). These agents are thought to act on intrinsic neurons to stimulate gastric, small bowel, and colonic transit in health, in constipation and in IBS with constipation (Bouras et al., 1999; Poen et al., 1999; Degen et al., 2001). In the stomach, 5-HT_4_ receptor agonists enhance (postprandial) proximal gastric volumes in health, but have no effects on sensation (Tack et al., 2003). Prucalopride was also shown to be effective in the treatment of constipation (Emmanuel et al., 2002).

### Linaclotide

This is an example of guanylate cyclase-C agonist (Busby et al., 2013), which has been shown to reduce visceral hypersensitivity in preclinical studies and to improve abdominal pain and constipation symptoms in phase 2 and 3 clinical trials of patients with IBS and constipation (Johnston et al., 2013).

### Lubiprostone

This is an oral bicyclic fatty acid selectively activating type 2 chloride channels in the apical membrane of the intestinal epithelial cells, hence stimulating chloride secretion, along with passive secretion of sodium and water, inducing peristalsis and laxation, without stimulating gastrointestinal smooth muscle (Schey and Rao, 2011). It is indicated in IBS with constipation. Considering the importance of epithelial barrier function and cell integrity and the known impact of stressors, the observation that lubiprostone exhibits the additional distinct property of effective protection or repair of the epithelial barrier and cell function after stress suggests potential clinical importance for patients with impaired barrier function, which might occur in IBS (Cuppoletti et al., 2012).

### Tachykinin receptor antagonists

Three distinct receptors, NK_1_, NK_2_, and NK_3_, mediate the biological effects of endogenous tachykinins SP, NKA, and NKB, in the gastrointestinal tract. Through the locations of NK receptors on intrinsic nerves, extrinsic nerves, inflammatory cells, and smooth muscle, inhibition of tachykinin receptors has the potential to inhibit motility, sensitivity, secretion, and inflammation in the gastrointestinal tract (Holzer, 2004a; Lecci et al., 2004). NK_1_ receptor antagonists also have antiemetic properties (Holzer, 2004a). Several tachykinin receptor antagonists have been developed so far, but the results, in general, have been disappointing. Recently, chronic treatment with AV608 (NK_1_ receptor antagonist) in IBS has been reported to be associated with improved mood and pain ratings and activity of emotional arousal related brain regions (Tillisch et al., 2012).

### Adrenoceptor agonist

*The α_2_-adrenoceptor agonist* clonidine was shown to reduce colonic tone and pain sensation in response to distension (Bharucha et al., 1997; Malcolm et al., 2000; Viramontes et al., 2001b). A preliminary study of clonidine in IBS with diarrhea suggested therapeutic potential for clonidine, but clinical application is hampered by dose-limiting side-effects like somnolence or hypotension. Among β_3_-adrenoceptor agonists, solabegron is an example (Grudell et al., 2008).

### Opioid receptor ligands

Three types of opioid receptors, μ-, δ-, and κ-receptors, located in the ENS as well as on nociceptive pathways, have effects on human gastrointestinal function. Opioid receptor activation reduces visceral pain through peripheral (spinal afferents) and central mechanisms, and inhibits motility through decreased acetylcholine release. κ-Opioid receptor agonists have been proposed as a pharmacological approach to the treatment of altered visceral sensitivity. Acute studies with fedotozine and asimadoline showed decreased sensitivity to gastric or colonic distension (Coffin et al., 1996; Delvaux et al., 1999, 2002; Delgado-Aros et al., 2003). However, therapeutic studies in IBS and FD with fedotozine have been disappointing (Dapoigny et al., 1995; Read et al., 1997). The μ-opioid receptor agonist loperamide, used in the treatment of diarrhea, inhibits secretion, reduces colonic transit, and increases resting anal sphincter tone (Corazziari, 1999). Peripherally acting μ-opioid receptor antagonists (e.g., N-methylnaltrexone and alvimopan) normalize bowel function in opiate-treated patients without compromising central opioid analgesia (Holzer, 2004b). Racecadotril, a neutral endopeptidase (NEP) inhibitor, increases the exposure to NEP substrates including enkephalins: it was found consistently effective in animal models and patients with various forms of acute diarrhea by inhibiting secretion from the gut without changing gastrointestinal transit time or motility (Eberlin et al., 2012). In direct comparative studies with loperamide, racecadotril was at least as effective, but exhibited fewer adverse events in most studies, particularly less rebound constipation (Eberlin et al., 2012). However, its potential in IBS remains to be established.

### Miscellaneous agents

CCK has a large number of effects on gastrointestinal contractility and secretion (Walsh, 1994). CCK1 receptor antagonists like loxiglumide and dexloxiglumide enhance gastric emptying in health and in IBS with constipation, though effects on colonic motility are unclear (De Ponti and Malagelada, 1998; Scarpignato and Pelosini, 1999) and clinical usefulness has not been established.

The transient receptor potential ion channel of the *vanilloid type 1 (TRPV1)*, expressed by primary afferent neurons, is viewed as a trigger for chemonociception and may be upregulated in some functional gut disorders (Chan et al., 2003). TRPV1 and TRPV4 blockade are areas of current investigation (Holzer, 2011; Fichna et al., 2012).

*Muscarinic receptor antagonists* and *smooth muscle relaxants* are used in some countries for the treatment of IBS. Meta-analysis suggests they are superior to placebo in IBS-related pain (Poynard et al., 2001), though the quality of trials is often questionable.

*Cannabinoid* CB_1_ receptors are expressed on nociceptive afferents and ENS neurons while CB_2_ receptors are expressed on immune cells (Schicho and Storr, 2011). Activation of CB_1_ receptors slows gastrointestinal transit in animals through inhibition of acetylcholine release. The non-specific agonist delta-9-tetrahydrocannabinol has strong antiemetic properties and delays gastric emptying in humans (Frytak et al., 1979; McCallum et al., 1999). The observation that, in an animal model of intestinal inflammation, CB_1_ and CB_2_ receptor subtypes are upregulated opens a new perspective on the possible use of CB_1_ or CB_2_ receptor agonists in postinfectious IBS with diarrhea (Kimball et al., 2010). Indeed, dronabinol, a non-selective CB agonist, reduces fasting colonic motility in patients with IBS with diarrhea or alternating (Wong et al., 2011).

Finally, α2δ ligands such as gabapentin and pregabalin (Gale and Houghton, 2011) have undergone a number of preclinical and clinical tests for their potential in disorders with visceral hypersensitivity. In fact, voltage-sensitive Ca^2+^ channels are involved in neural function and have an α2δ binding site to which the aforementioned ligands bind potently, reducing Ca^2+^ influx at the nerve terminals. Pregabalin was effective in several animal models of visceral pain (Gale and Houghton, 2011).

## Conclusion

From the above overview, it is clear that research in the treatment of IBS is still very active. Although in the past decade some innovative pharmacological agents have not fulfilled their promise because of unexpected side-effects, it is likely that new pathophysiological concepts as well as the publication of new regulatory documents by the FDA and the EMA will be of great help for drug developers.

## Conflict of Interest Statement

The author declares that the research was conducted in the absence of any commercial or financial relationships that could be construed as a potential conflict of interest.

## Abbreviations

EMA, European Medicines Agency; ENS, enteric nervous system; FDA, food and drug administration; 5-HT, 5-hydroxytryptamine; IBS, irritable bowel syndrome.
